# Finding the hiding spots: uneven distribution of pathogenic *Leptospira* spp. in the bovine genital tract

**DOI:** 10.3389/fpubh.2025.1735321

**Published:** 2026-01-12

**Authors:** Paulo Victor dos S. Pereira, Ana Paula da S. Cupello, Lucas Francisco L. Correia, Walter Lilenbaum, Joanna M. G. Souza-Fabjan

**Affiliations:** 1 Faculdade de Veterinária, Universidade Federal Fluminense, Niterói, Brazil; 2 Laboratório de Bacteriologia Veterinária, Instituto Biomédico, Universidade Federal Fluminense, Niterói, Brazil

**Keywords:** leptospirosis, reproductive tract, reproductive failure, uterus, zoonosis

## Abstract

**Background:**

Bovine genital leptospirosis (BGL) is a chronic reproductive disease caused by pathogenic *Leptospira* spp., whose uneven distribution in the genital tract may compromise diagnosis.

**Objective:**

This study evaluated the presence of pathogenic *Leptospira* spp. in different regions of the bovine reproductive tract of naturally infected cows to identify the most reliable anatomical site for molecular detection.

**Methods:**

Oviducts (OVID) and uterine fragments from the uterine body (UB), base of the uterine horn (BUH), and apex of the uterine horns (AUH) were collected *post-mortem* from 40 cows.

**Results:**

Pathogenic *Leptospira* spp. DNA was detected by lip*L32* PCR in at least one anatomical site in 55% (22/40) of animals. The highest positivity rate was observed in UB (20/40; 50%), whereas AUH (7.5%), BUH (0%), and OVID (2.5%) showed no or minimal detection.

**Conclusion:**

The findings demonstrate that pathogenic *Leptospira* spp. are unevenly distributed within the bovine reproductive tract, with a clear preference for the uterine body. This anatomical site provides the greatest diagnostic accuracy and should be prioritized for molecular testing to minimize false-negative results and improve BGL surveillance.

## 1 Introduction

Leptospirosis is a globally distributed zoonotic disease caused by pathogenic bacteria of the genus *Leptospira*. In cattle, it can manifest as bovine genital leptospirosis (BGL), a chronic and often subclinical condition that compromises reproductive performance. The primary reproductive disorders associated with BGL include embryonic loss, infertility, and early fetal death (1). Leptospirosis represents not only a veterinary concern but also a major zoonotic threat within the One Health and Planetary Health frameworks, as it emerges at the interface between livestock, wildlife, and environmental systems. Understanding its persistence in animal hosts is, thus, critical for preventing transmission to humans and reducing the ecological burden of this neglected disease.

Previous studies have demonstrated the presence of pathogenic *Leptospira* spp. in various sites of the bovine reproductive tract, including the uterus (2), cervicovaginal mucus (3), ovaries and oviducts (4), follicular fluid (5), and cumulus–oocyte complexes (13). These findings support the hypothesis that genital colonization facilitates the silent persistence and dissemination of the pathogen within herds, especially when conventional tests based exclusively on urine samples are used, leading to underdiagnosis of BGL (6).

In addition to its epidemiological impact, uterine colonization by pathogenic *Leptospira* spp. may cause microenvironmental alterations that impair embryo development. Pedrosa et al. (7) detected pathogenic *Leptospira* DNA in uterine tissues and reported increased interleukin-6 (IL-6) concentrations in positive samples, suggesting that leptospiral colonization triggers a local inflammatory response capable of compromising uterine receptivity. Such chronic inflammation may contribute to embryo loss and irregular estrous cycles in affected females, as observed in the low fertility rates of infected cows.

Regarding diagnostic approaches, cervicovaginal mucus sampling and *in vivo* uterine biopsies have been proposed as effective and welfare-compatible alternatives for live animals (8). Later on, Aymée et al. (6) demonstrated that both cervicovaginal mucus and uterine fragments (UF) yield comparable results for detecting pathogenic *Leptospira* spp. in naturally infected cows, with positivity rates markedly higher than those observed in urine. These findings confirm that genital tract samples are more reliable for molecular diagnosis of BGL and highlight the diagnostic value of direct tissue sampling, which enhances test sensitivity and minimizes false negatives caused by intermittent bacterial shedding. Nevertheless, it remains unclear whether pathogenic *Leptospira* spp. are evenly distributed within the genital tract, which could influence the diagnostic sensitivity of tissue biopsies depending on the anatomical site. To date, no study has compared the detection of pathogenic *Leptospira* spp. among distinct genital regions or assessed whether a specific site better represents the infection status of the entire system.

Mapping the anatomical niches that sustain pathogenic *Leptospira* persistence contributes to integrated surveillance strategies, particularly in tropical regions where environmental and climatic drivers favor pathogen maintenance and dissemination. Understanding the spatial distribution of pathogenic *Leptospira* spp. within the female reproductive tract is therefore essential for optimizing sampling protocols and reducing false-negative results. Hence, the objective of this study was to investigate the presence of *Leptospira* spp. DNA in different anatomical regions of the genital tract—oviducts and distinct uterine sites—of naturally infected cows.

## 2 Method

### 2.1 Location, and sample collection

This study used *post-mortem* samples from crossbreed bovine reproductive tracts donated by a commercial slaughterhouse in Rio de Janeiro, Brazil. Therefore, ethical approval was not required by the Animal Use Ethics Committee.

Oviducts (OVID) and fragments from the uterine body (UB), base of the uterine horn (BUH), and apex of the uterine horn (AUH) were aseptically collected from 40 non-pregnant cows immediately after slaughter. For aseptic collection, the gloves, tweezers, and surgical scissors used were disinfected with 70° GL alcohol after each sample collected. Samples from each animal were stored individually in sterile conical tubes to prevent cross-contamination and transported to the laboratory at 4 °C. The OVID samples were used in their totality, while uterine fragments were processed by dissecting the endometrium and myometrium. All samples were stored at −20 °C until molecular detection of pathogenic *Leptospira* spp. by conventional lip*L32* PCR (cPCR). Samples testing negative by cPCR were further analyzed by quantitative lip*L32* PCR (qPCR) to confirm results.

### 2.2 Molecular diagnosis

DNA from OVID and uterine samples was extracted from 25 mg of tissue per sample using the DNeasy® Blood & Tissue Kit (Qiagen, California, USA), following the manufacturer’s instructions. Conventional PCR targeting the lip*L32* gene, present exclusively in pathogenic *Leptospira* species, was performed as described by Hamond et al. (9). The concentration of primers used was 0.6 μM, 1.0 U Taq polymerase, 2.4 μM MgCl_2_, and 0.3 mM dNTP in a final volume of 25 μL. A denaturation cycle was first performed at 94 °C for 2 min, followed by 35 cycles of denaturation at 94 °C for 30 s, annealing at 53 °C for 30 s, extension at 72 °C for 1 min, and a final extension at 72 °C for 5 min. DNA from *L. interrogans* serovar Copenhageni (Fiocruz L1-130) served as the positive control, and ultrapure water as the negative control. Although a specific internal inhibition control was not included in the reactions, all samples were processed with standard purification steps validated for reproductive tissues, and negative controls showed no abnormal amplifications. The PCR products were analyzed under UV light after resolution on a 2% agarose gel electrophoresis stained with GelRed. The diagnosis for BGL was confirmed once an animal presented a positive *lip*L32 cPCR result in any sample. Quantitative PCR (qPCR) was performed according to Stoddard (10), using the Amplio Mastermix qPCR 2 × MDx Kit (Loccus, Cotia, SP, Brazil), primers 45F (5′-AAG CAT TAC CGT TGT GGT G-3′) and 286R (5′-GAA CTC CCA TTT CAG CGA TT-3′), and a probe (5′−/56-FAM/AAA GCC AGG ACA AGC GCC G/3BHQ_1/−3′), following the manufacturer’s guidelines. Although the detection threshold was not experimentally determined in our laboratory, the qPCR assay used has a reported analytical sensitivity of 1–10 genome equivalents per reaction (10).

### 2.3 Statistical analysis

Categorical data were analyzed by Fisher’s exact test to assess possible associations between infection status and anatomical sites. The comparison among collection sites was performed using Cochran’s Q test. All analyses were performed in SPSS 27 Statistics Base Software (IBM), with a 95% confidence interval, and a significance level of *p* < 0.05.

## 3 Results

**Figure 1:**
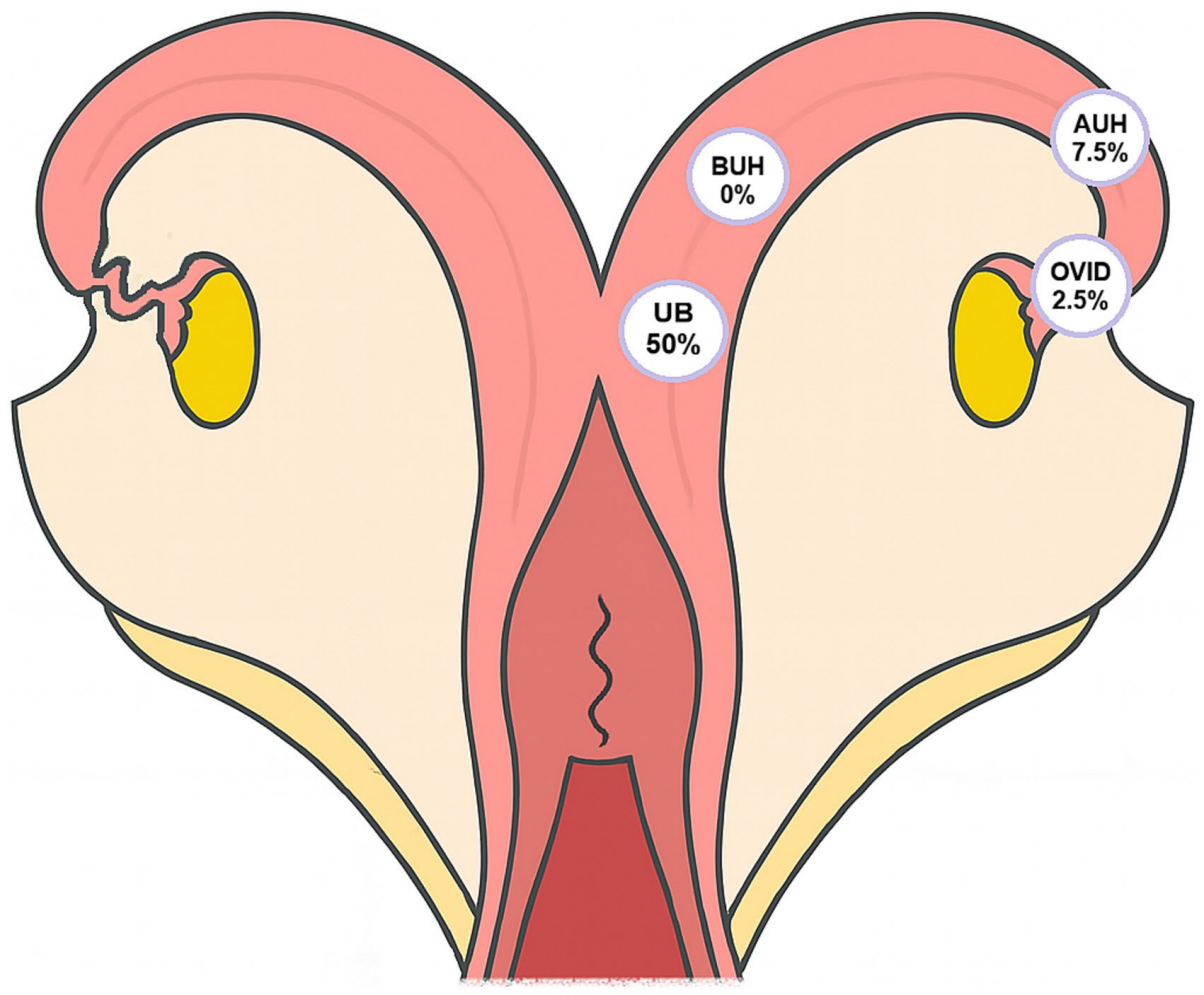
Detection of *Leptospira* spp. by PCR in different anatomical sites of the bovine female genital tract in naturally infected cows. Percentages indicate the proportion of PCR-positive samples obtained from 25 mg of tissue per site, based on samples collected from 40 cows. Uterine (uterine body and horns) and oviductal segments are shown.

Considering the overall infection status, here defined as the detection of pathogenic *Leptospira* DNA in at least one of the analyzed anatomical sites, 55% (22/40) of the cows were classified as positive for BGL. Among the specific regions examined, pathogenic *Leptospira* DNA was detected in only one oviduct sample (2.5%; *p* = 0.550), whereas uterine fragments showed markedly different results: 50% (20/40; *p* < 0.001) of the UB samples were positive, while no positive results were found in the BUH; 0%; *p* = 1.0 and only 7.5% (3/40; *p* = 0.156) in the AUH (Figure 1). Statistical comparison among paired collection sites confirmed that the UB presented significantly higher positivity (*p* < 0.01) than all the other regions, which did not differ from each other (*p* = 1.0). The results obtained are presented in Table 1.

**Table 1:** Individual PCR results for *Leptospira* spp. detection across anatomical sites of the bovine female genital tract in naturally infected cows.

	Molecular status by anatomical site				
Cow	UB	BUH	AUH	OVID	Overall
1	+	−	+	−	+
2	−	−	−	−	−
3	+	−	−	−	+
4	+	−	−	−	+
5	+	−	−	−	+
6	−	−	−	−	−
7	+	−	−	−	+
8	−	−	+	−	+
9	−	−	−	−	−
10	+	−	−	−	+
11	+	−	−	−	+
12	+	−	−	−	+
13	−	−	−	−	−
14	−	−	−	−	−
15	−	−	−	−	−
16	+	−	−	−	+
17	+	−	−	−	+
18	−	−	−	−	−
19	+	−	−	−	+
20	−	−	−	−	−
21	+	−	−	−	+
22	−	−	−	−	−
23	−	−	+	−	+
24	−	−	−	−	−
25	−	−	−	−	−
26	−	−	−	−	−
27	+	−	−	−	+
28	−	−	−	−	−
29	+	−	−	−	+
30	+	−	−	+	+
31	+	−	−	−	+
32	+	−	−	−	+
33	−	−	−	−	−
34	+	−	−	−	+
35	+	−	−	−	+
36	−	−	−	−	−
37	+	−	−	−	+
38	−	−	−	−	−
39	−	−	−	−	−
40	−	−	−	−	−

UB, uterine body; BUH, base of the uterine horn; AUH, apex of the uterine horn; OVID, oviducts; +, Positive; −, Negative.

## 4 Discussion

This study reinforces the relevance of BGL as a widespread reproductive infection in female cattle. The high overall positivity rate (55%) observed in slaughtered cows indicates a high prevalence of pathogenic *Leptospira* spp. in the reproductive tract, in agreement with previous reports identifying the pathogen in uterine tissue and cervicovaginal mucus of subfertile cows (2, 11). These findings support the notion that genital colonization represents an important mechanism of pathogen persistence within cattle populations and may contribute to underdiagnosis when surveillance relies solely on conventional clinical or urinary assessments. From a One Health perspective, the persistence of leptospires in reproductive tissues represents a potentially underestimated interface between livestock production, environmental contamination, and zoonotic risk.

The most important outcome of the study was the markedly high detection rate observed in the UB (50%), supporting the hypothesis that this region constitutes a key anatomical site of pathogenic *Leptospira* colonization in BGL. Similar observations have been reported by Pires et al. (12) and Pedrosa et al. (7), who identified that uterine infection activates local inflammatory pathways, increasing cytokine levels and possibly compromising embryo viability. Together, these data reinforce the biological relevance of uterine colonization beyond its diagnostic implications.

In contrast, no positive samples were found in the BUH, and only low detection rates occurred in the AUH (7.5%) and OVID (2.5%). This uneven distribution suggests site-specific differences in susceptibility to colonization within the female genital tract, likely driven by a combination of anatomical and physiological factors. In addition to the higher vascularization of the UB, luminal fluid dynamics, hormonal fluctuations across the estrous cycle, and regional differences in epithelial architecture and receptivity may modulate bacterial persistence and detection, as previously suggested (7). From a diagnostic perspective, this anatomical asymmetry implies that sampling restricted to distal uterine sites may result in false-negative outcomes, reinforcing the UB as the most reliable anatomical site for molecular detection.

The interpretation of these findings must consider the inherent limitations associated with the use of slaughterhouse-derived samples. The cows included in this study were slaughtered for unknown reasons, and no clinical, reproductive, or serological histories were available. Consequently, it was not possible to distinguish between asymptomatic animals, cows with subclinical reproductive impairment, or animals removed from production for reasons unrelated to reproduction. While this limitation precludes definitive conclusions regarding the clinical expression of infection at the individual level, the frequent detection of pathogenic *Leptospira* spp. in reproductive tissues underscores the potential for genital colonization to occur without overt clinical information at the time of slaughter, highlighting a critical gap in routine surveillance strategies.

Although the UB was identified as the most consistent site for molecular detection, it is important to acknowledge that not all tissues analyzed in this *post-mortem* study are readily accessible under field conditions. In live animals, uterine sampling is feasible primarily through transcervical biopsy, a technique previously described as applicable for the diagnosis of BGL (6). Cervicovaginal mucus remains the least invasive and most commonly used alternative in routine practice (6, 11). In this context, the present findings suggest that, whenever uterine sampling is performed, prioritizing regions anatomically closer to the UB may improve diagnostic sensitivity and reduce false-negative outcomes associated with the asymmetric distribution of leptospires within the uterus.

There are additional limitations to be considered. Although the overall prevalence observed in this study falls within the range reported in previous investigations conducted in the same geographical region (4, 5, 11), the sample size remains limited for drawing broader epidemiological inferences. In particular, the low number of positive samples in specific anatomical sites may have reduced the statistical power to detect subtle regional differences. Moreover, information on breed composition, farm management practices, and/or the level of technological development was not available, limiting the generalizability of the findings to other production systems. In a previous study, crossbred pluriparous cows from medium- to high-technification commercial dairy herds were used, and the presence of pathogenic *Leptospira* spp. was detected in approximately 27% of the animals (4). Finally, the detection threshold of the qPCR assay was not experimentally determined, precluding quantitative comparisons of bacterial load among samples, including cases in which multiple anatomical sites tested positive.

In conclusion, this study demonstrates that pathogenic *Leptospira* spp. are unevenly distributed within the bovine female reproductive tract, with a marked predominance in the UB. These findings suggest that BGL may frequently remain undetected under field conditions, particularly in the absence of overt clinical or reproductive data, although the lack of individual animal histories limits definitive conclusions regarding subclinical infection. Collectively, these results strengthen our understanding of BGL pathophysiology and reinforce the importance of targeted sampling strategies to improve diagnosis, surveillance, and control in cattle production systems. These results not only enhance diagnostic precision in bovine leptospirosis but also contribute to a broader understanding of how pathogen persistence in livestock can influence zoonotic risks and ecosystem health under the One Health framework.

## Data Availability

The original contributions presented in the study are included in the article/supplementary material, further inquiries can be directed to the corresponding author/s.
